# Supplementary material for: Comparative Genomics and Functional Studies of Putative m6A Methyltransferase (METTL) Genes in Cotton
Source: Int J Mol Sci. 2022 Nov 15;23(22):14111. doi: 10.3390/ijms232214111 (PMC9694044; doi:10.3390/ijms232214111)
Supplement: Supplementary file 1 [file ijms-23-14111-s001.zip › Figure S8.pdf]

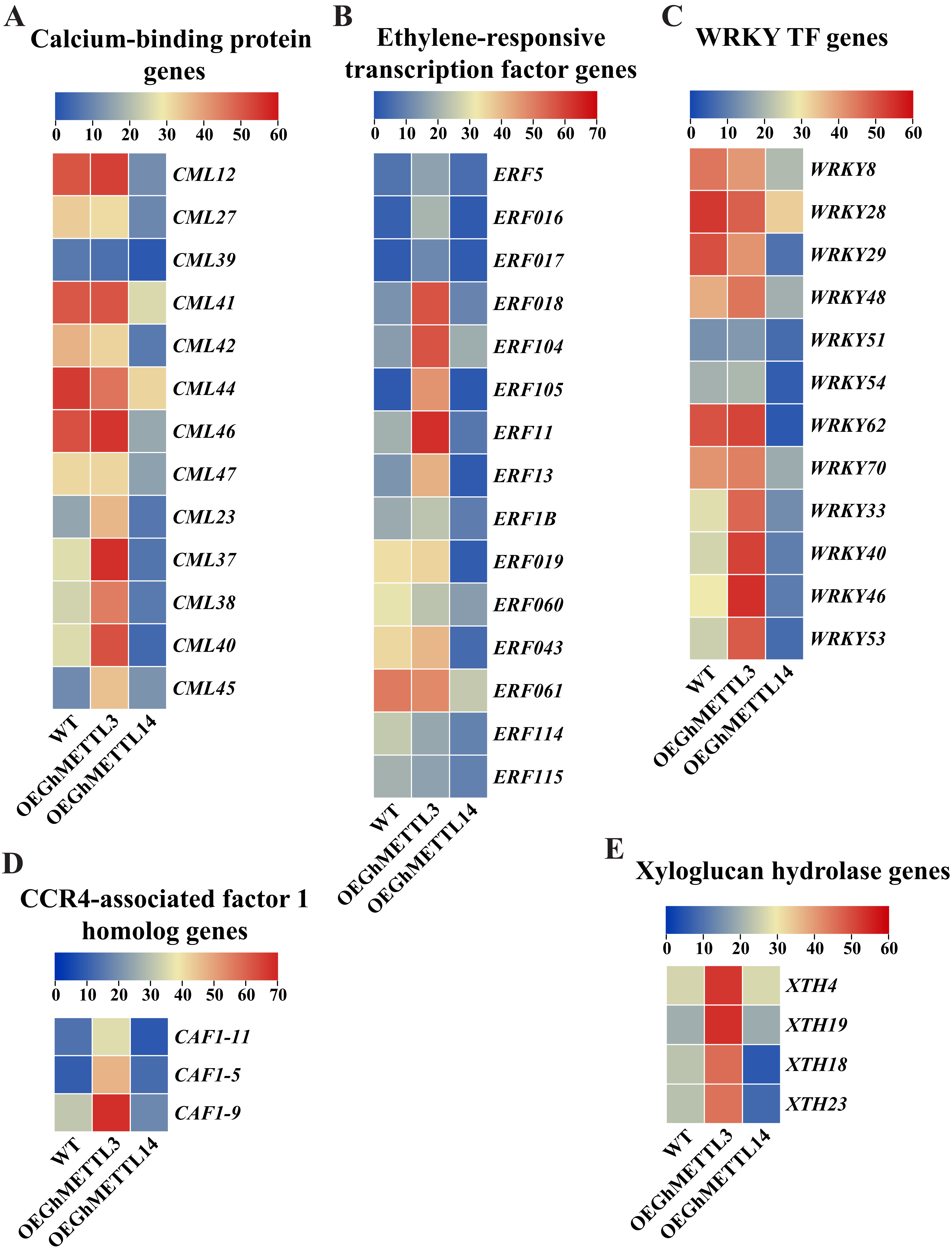
A
Calcium-binding protein genes
0 10 20 30 40 50 60
CML12
CML27
CML39
CML41
CML42
CML44
CML46
CML47
CML23
CML37
CML38
CML40
CML45
WT
OEGhMETTL3
OEGhMETTL14
B
Ethylene-responsive transcription factor genes
0 10 20 30 40 50 60 70
ERF5
ERF016
ERF017
ERF018
ERF104
ERF105
ERF11
ERF13
ERF1B
ERF019
ERF060
ERF043
ERF061
ERF114
ERF115
WT
OEGhMETTL3
OEGhMETTL14
C
WRKY TF genes
0 10 20 30 40 50 60
WRKY8
WRKY28
WRKY29
WRKY48
WRKY51
WRKY54
WRKY62
WRKY70
WRKY33
WRKY40
WRKY46
WRKY53
WT
OEGhMETTL3
OEGhMETTL14
D
CCR4-associated factor 1 homolog genes
0 10 20 30 40 50 60 70
CAF1-11
CAF1-5
CAF1-9
WT
OEGhMETTL3
OEGhMETTL14
E
Xyloglucan hydrolase genes
0 10 20 30 40 50 60
XTH4
XTH19
XTH18
XTH23
WT
OEGhMETTL3
OEGhMETTL14
