# Supplementary material for: Comparative Genomics and Functional Studies of Putative m6A Methyltransferase (METTL) Genes in Cotton
Source: Int J Mol Sci. 2022 Nov 15;23(22):14111. doi: 10.3390/ijms232214111 (PMC9694044; doi:10.3390/ijms232214111)
Supplement: Supplementary file 1 [file ijms-23-14111-s001.zip › Table S1.pdf]

Table S1. List of forward and reverse primers used for this study.

Primer	Sequence (5'-3')	Purpose
Kan-F	GGCGATACCGTAAAGCACGAGGAA	Transgenic identification
Kan-R	GCTATGACTGGGCACAACAGACAAT	Transgenic identification
GhMETTL3-OE-F	ATGGAGAGCAACTCAGGCGG	Overexpression
GhMETTL3-OE-R	TCAGGTGGTCATATCAGTATCTAC	Overexpression
GhMETTL14-OE-F	ATGGATTCTCCTGAGCGTAGC	Overexpression
GhMETTL14-OE-R	CTACATTAAATTCATTGGTCTATG	Overexpression
GhMETTL3-VIGS-F	CGAACACGGTTAGTTCGAGA	VIGS
GhMETTL3-VIGS-R	GTCTCTGTGACGGCAAAAGG	VIGS
GhMETTL14-VIGS-F	AACAGATGGGCCTCCCAATG	VIGS
GhMETTL14-VIGS-R	TCTTTCAGGCGGTGTTCCTC	VIGS
GhMETTL3-RT-F	GCGGCAACCCATTGTTGATG	qRT-PCR
GhMETTL3-RT-R	AACCGTGTTCGGTCGATGC	qRT-PCR
GhMETTL14-RT-F	GGAGGAGGAGTTCTGGGGAT	qRT-PCR
GhMETTL14-RT-R	GCATGTCCTTTACCACCGGA	qRT-PCR
GhHIS3-RT-F	GAAGCCTCATCGATACCGTC	qRT-PCR
GhHIS3-RT-R	CTACCACTACCATCATGG	qRT-PCR
