# Supplementary figures and images for: Comparative Genomics and Functional Studies of Putative m6A Methyltransferase (METTL) Genes in Cotton
Source: Int J Mol Sci. 2022 Nov 15;23(22):14111. doi: 10.3390/ijms232214111 (PMC9694044; doi:10.3390/ijms232214111)

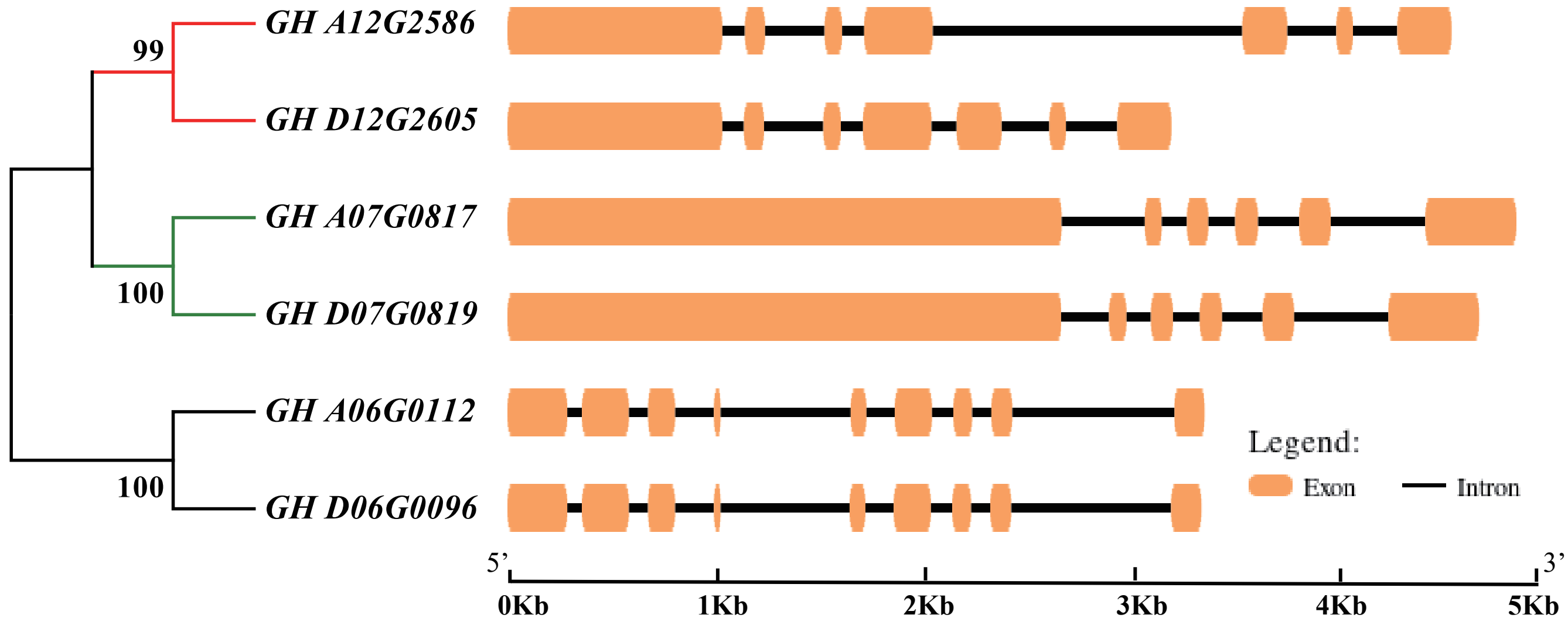

Supplement: Supplementary file 1 [file ijms-23-14111-s001.zip › Figure S1.pdf]

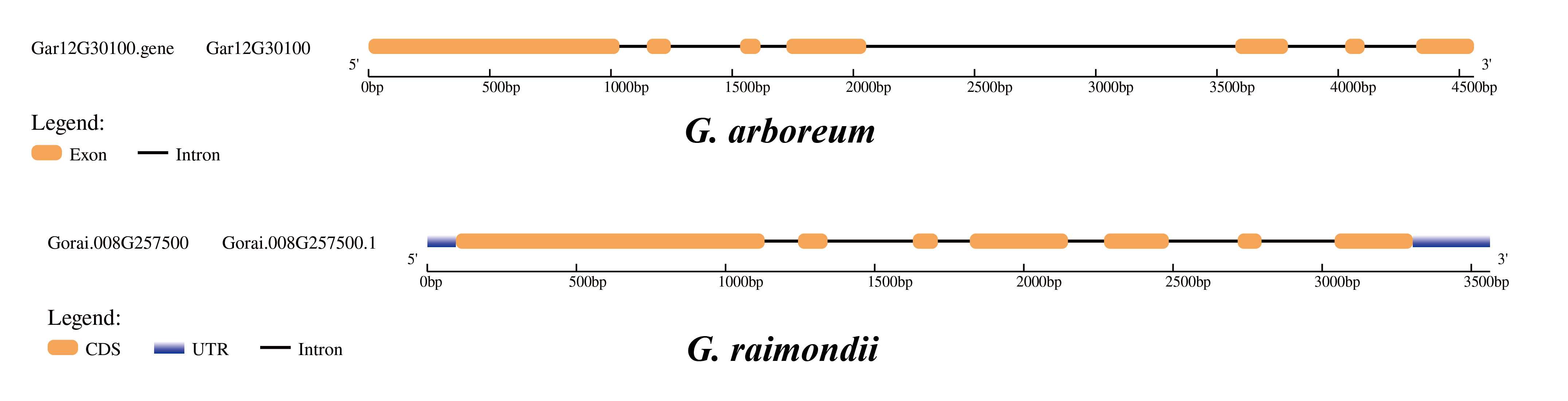

Supplement: Supplementary file 1 [file ijms-23-14111-s001.zip › Figure S2.jpg]

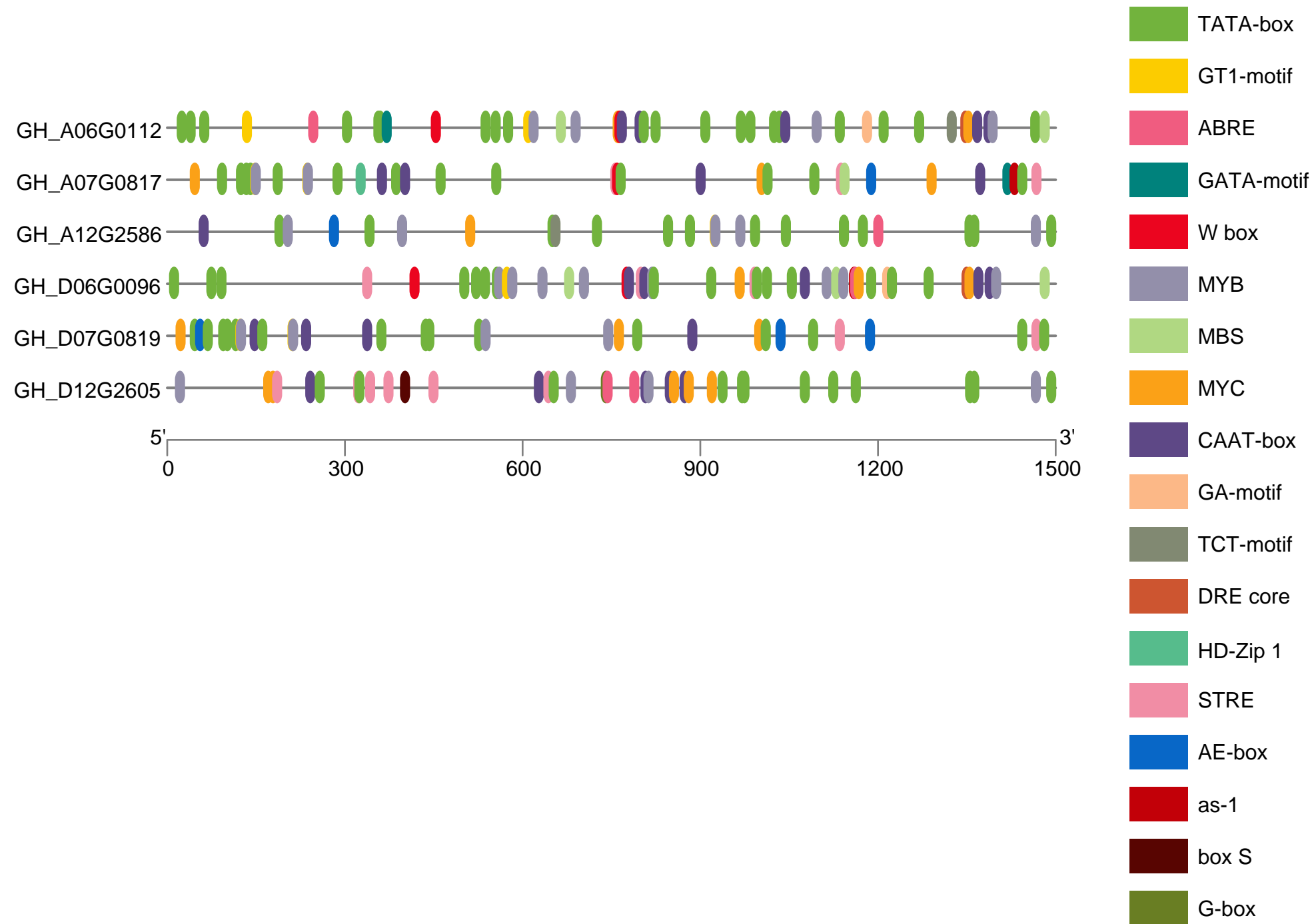

Supplement: Supplementary file 1 [file ijms-23-14111-s001.zip › Figure S3.pdf]

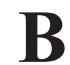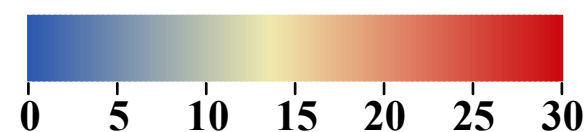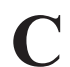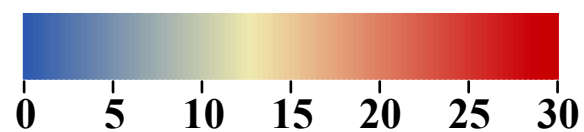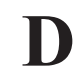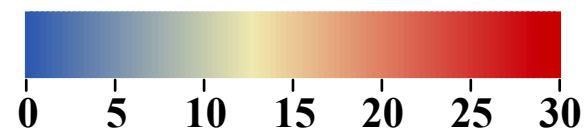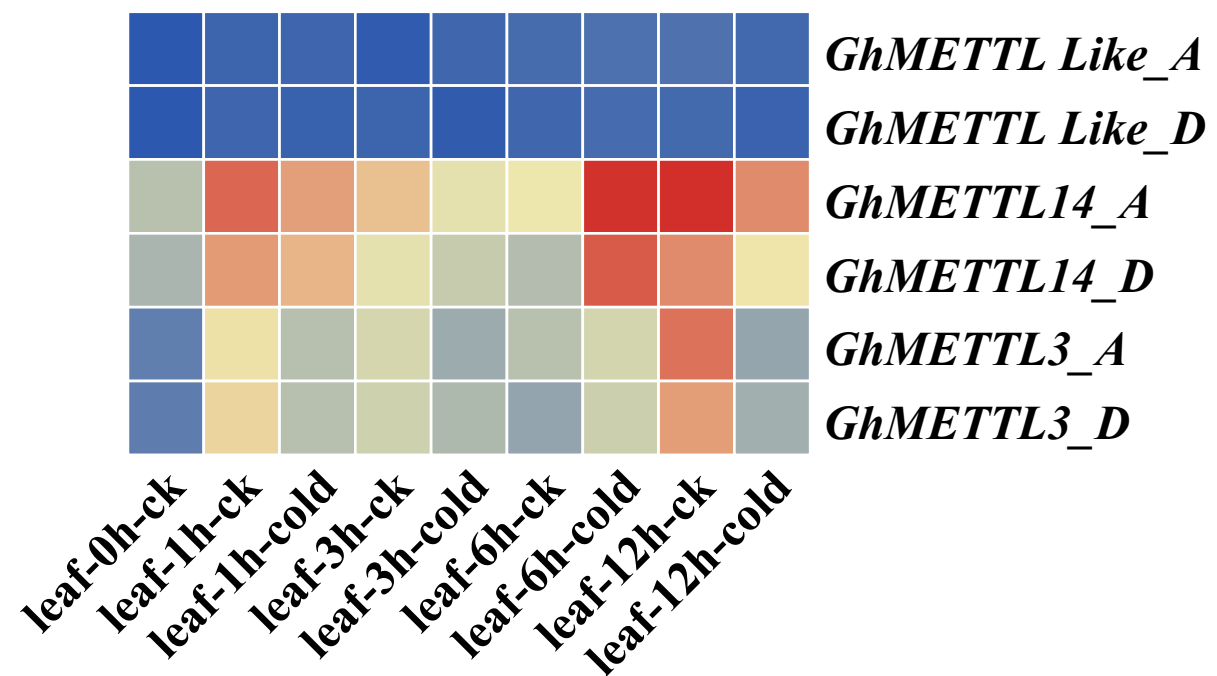

Supplement: Supplementary file 1 [file ijms-23-14111-s001.zip › Figure S4.pdf]

A

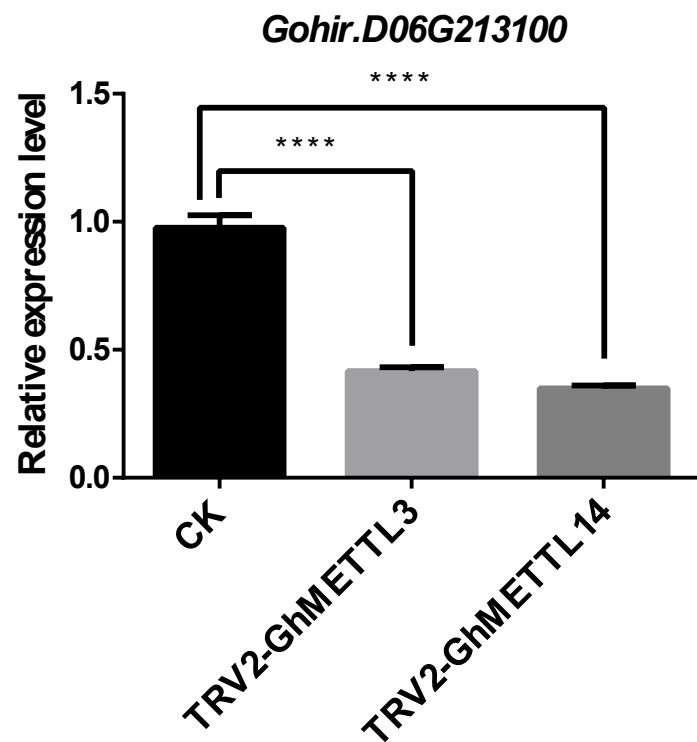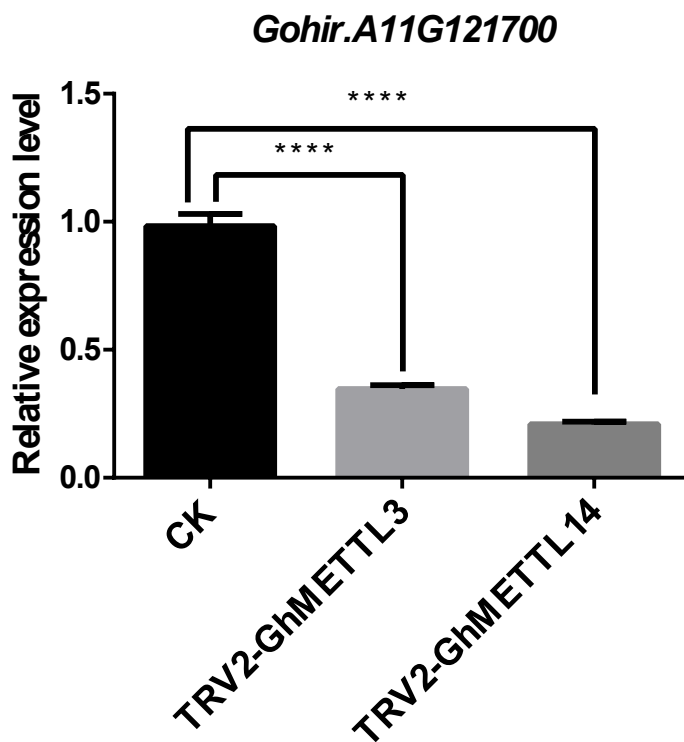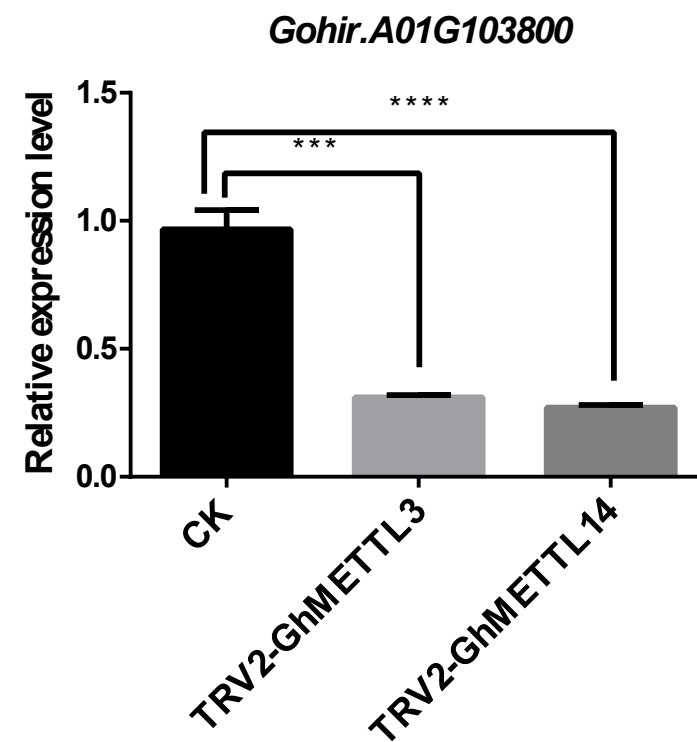

B

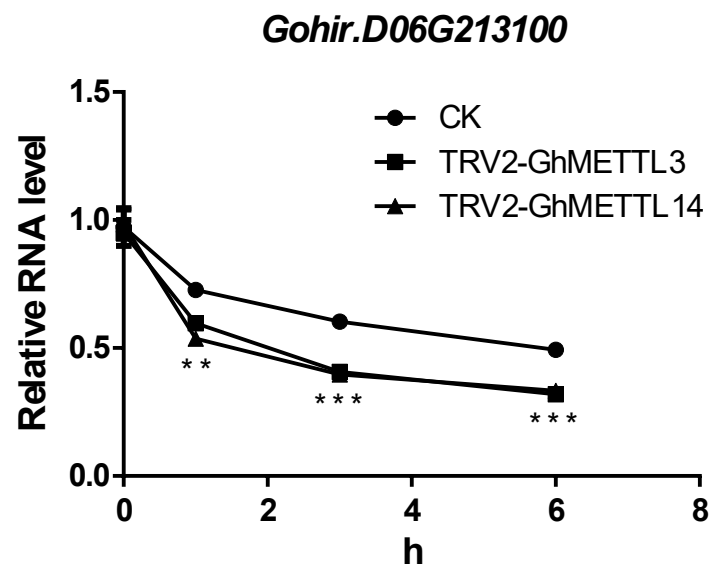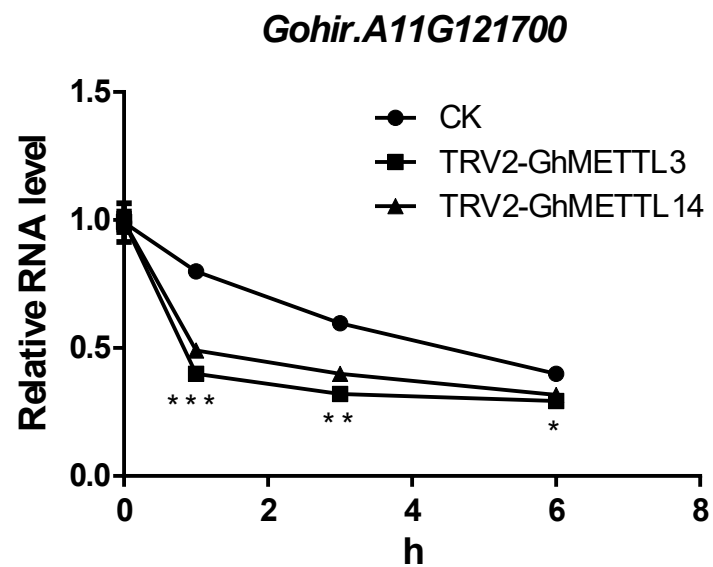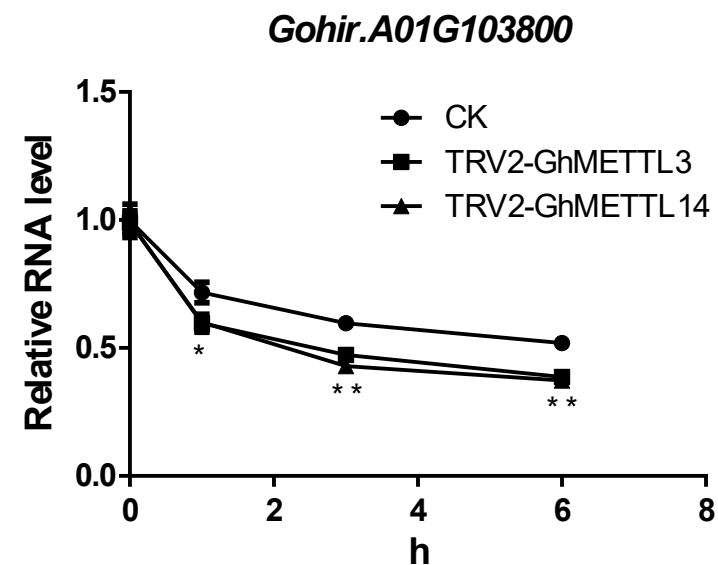

Supplement: Supplementary file 1 [file ijms-23-14111-s001.zip › Figure S5.pdf]

**A**

WT\_vs\_OEMTA.volcano

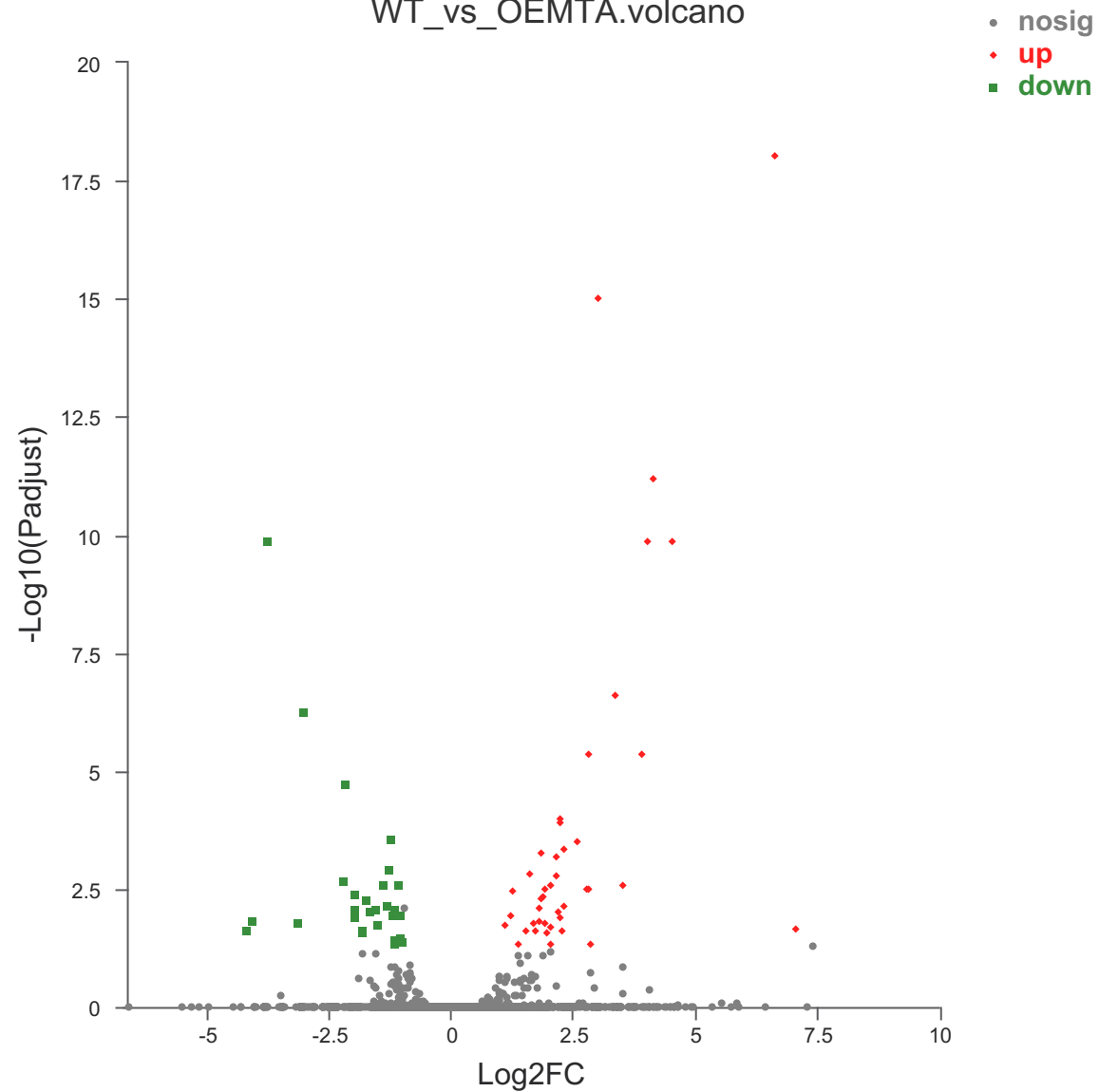**B**

WT\_vs\_OEMTB.volcano

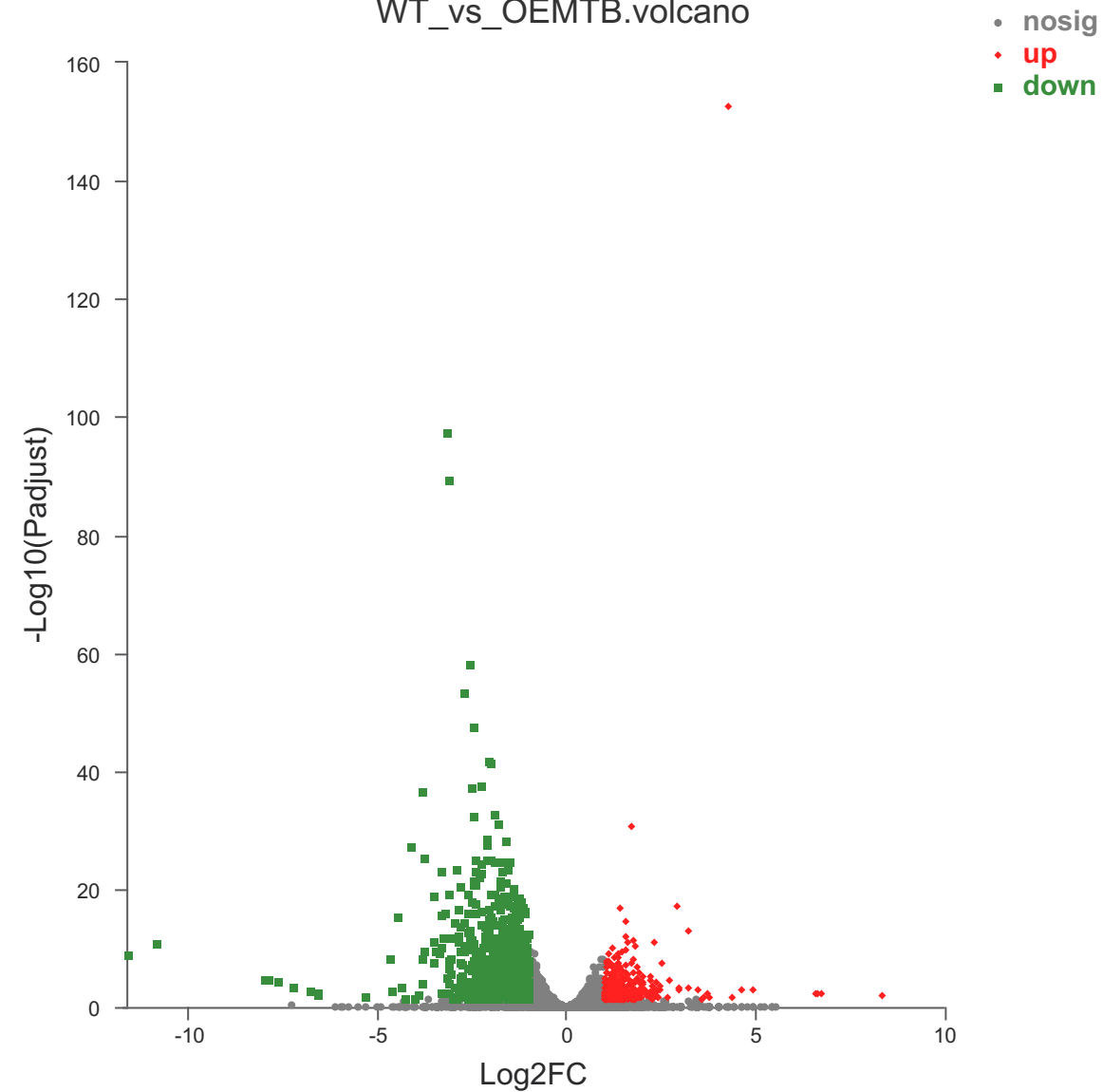

Supplement: Supplementary file 1 [file ijms-23-14111-s001.zip › Figure S6.pdf]

A

## KEGG enrichment analysis

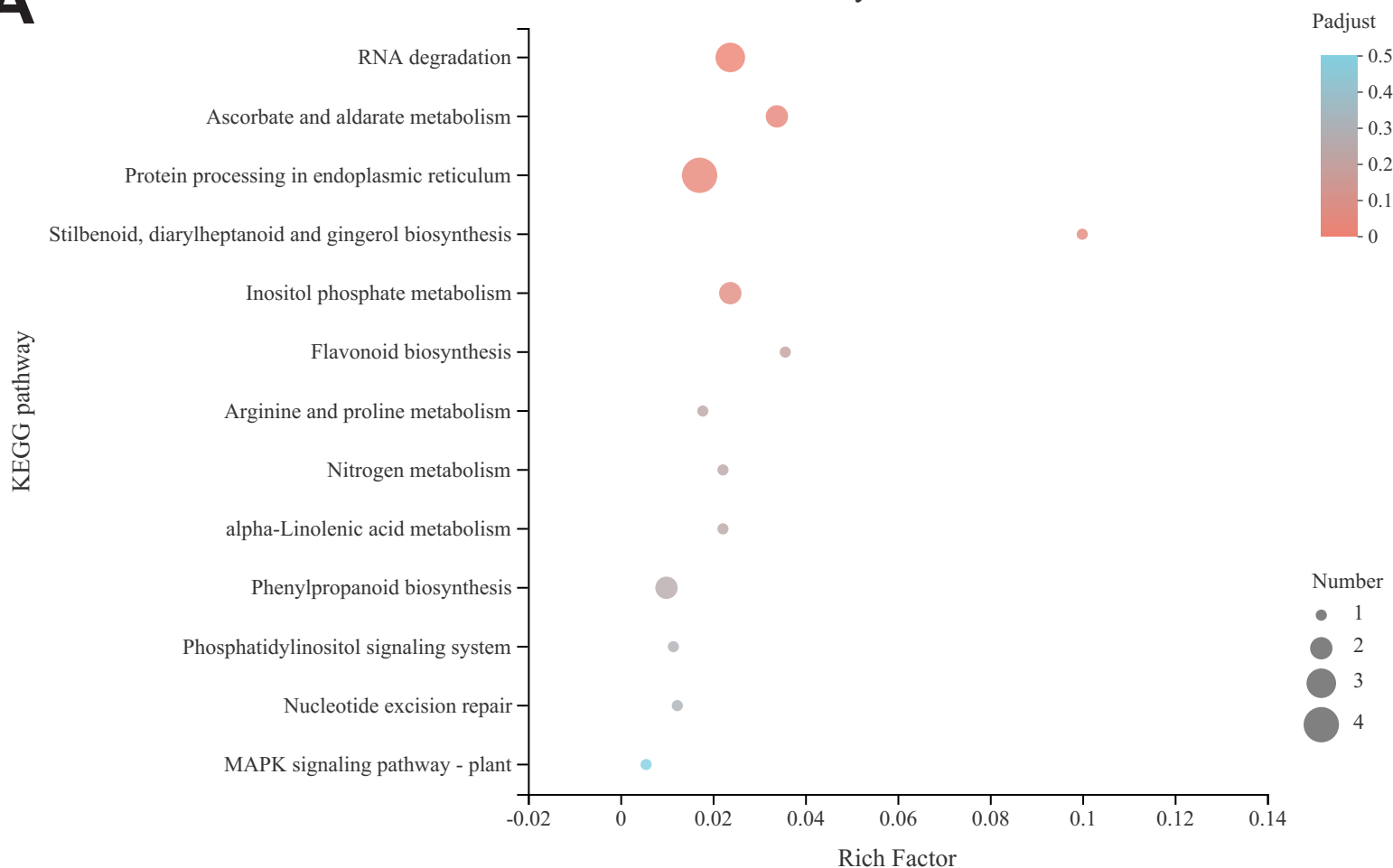

B

## KEGG enrichment analysis

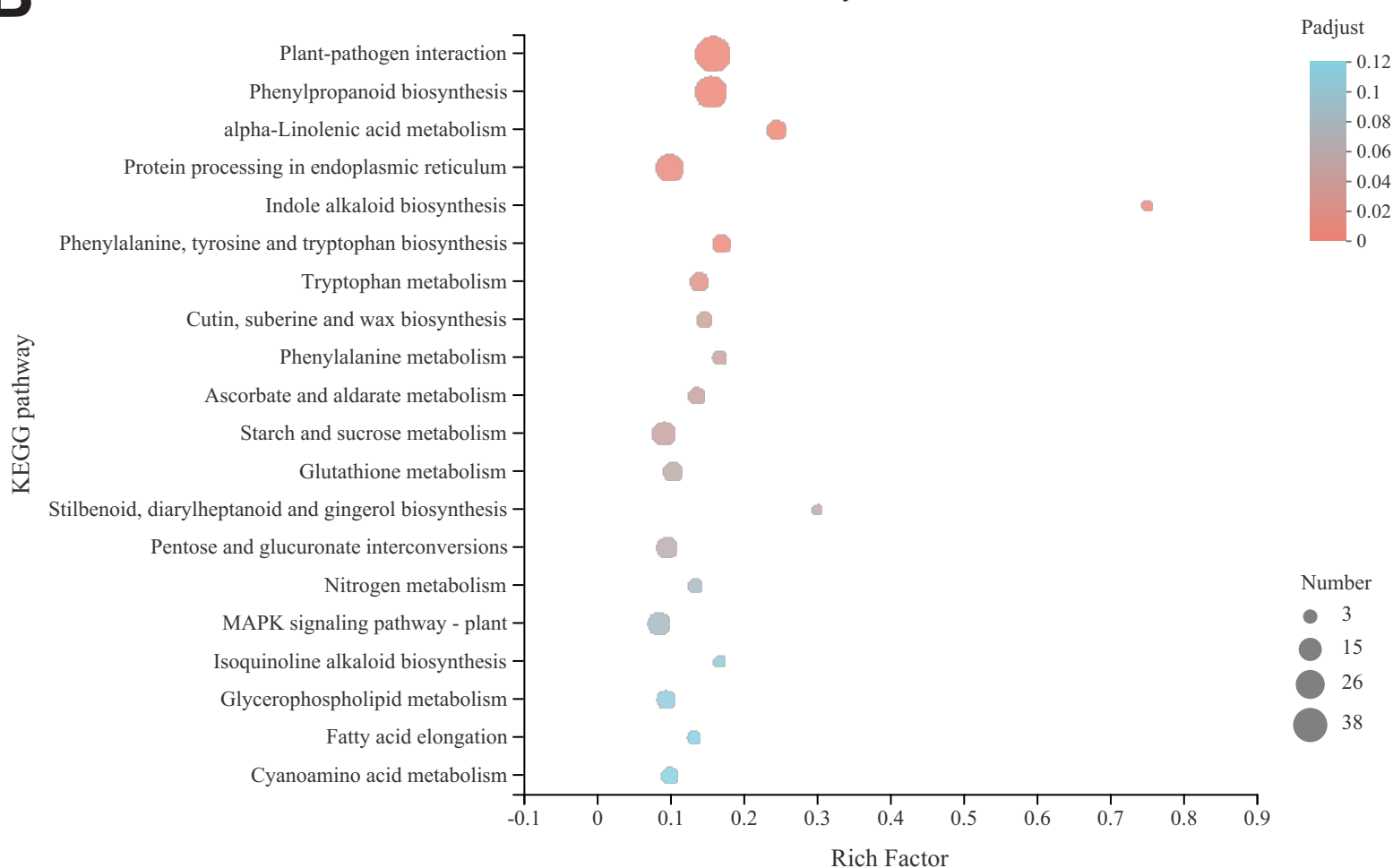

Supplement: Supplementary file 1 [file ijms-23-14111-s001.zip › Figure S7.pdf]
